# Preconceptional paternal alcohol consumption and the risk of child behavioral problems: a prospective cohort study

**DOI:** 10.1038/s41598-022-05611-2

**Published:** 2022-01-27

**Authors:** Min Luan, Xiaohua Zhang, Guanghong Fang, Hong Liang, Fen Yang, Xiuxia Song, Yao Chen, Wei Yuan, Maohua Miao

**Affiliations:** 1 NHC Key Laboratory of Reproduction Regulation (Shanghai Institute for Biomedical and Pharmaceutical Technologies), School of Public Health, Fudan University, Shanghai, 200237 China; 2 Minhang Maternal and Child Health Hospital, Shanghai, 201102 China; 3 NHC Key Laboratory of Reproduction Regulation (Shanghai Institute for Biomedical and Pharmaceutical Technologies), Fudan University, 779 Lao Hu Min Road, Shanghai, 200237 China

**Keywords:** Epidemiology, Paediatric research

## Abstract

Animal studies demonstrated that paternal alcohol exposure before conception increases the risk of adverse neurodevelopment in offspring, but limited evidence is known in humans. Based on Shanghai-Minhang Birth Cohort Study, we aimed to examine associations between preconceptional paternal alcohol consumption and child behavioral problems. Paternal alcohol consumption during the last 3 months before conception was obtained by maternal report. Children born to fathers who drank alcohol at least once a week were classified as exposed. Child behavioral problems were assessed using the Child Behavior Checklist (CBCL) at age of 2, 4, and 6. Negative binomial regression was used to estimate the rate ratio (RR) of CBCL raw scores in 796 offspring. The risks of rating scores on anxious/depressed were increased by 33% (RR 1.33, 95% CI 1.09, 1.61) and 37% (RR 1.37, 95% CI 1.02, 1.84) among boys in the exposed group at age of 4 and 6, respectively. We also found that risks of somatic complaints were increased by 18% (RR 1.18, 95%CI 1.00, 1.40) and 65% (RR 1.65, 95%CI 1.14, 2.38) among girls in the exposed group at age of 4 and 6. The increased risks of sleep problems (RR 1.25, 95% CI 1.00, 1.55) in girls at age 4, thought problems (RR 1.32, 95% CI 1.01, 1.73) in girls at age 6, rule-breaking behaviors (RR 1.35, 95% CI 1.09, 1.67) in boys at age 6 were also found. The risks of CBCL scores on anxious/depressed and sleep problems in girls at age 4, as well as the risks of somatic complaints and rule-breaking behaviors in boys at age 6 increased with the level of exposure to paternal alcohol consumption. Our findings provided preliminary evidence that preconceptional paternal alcohol consumption may increase risks of child behavioral problems.

## Introduction

It has been established that heavy maternal alcohol consumption during pregnancy can cause Fetal Alcohol Syndrome Disorders^1^, a syndrome characterized by physiological and neuropsychological deficits. In particular, emerging research has demonstrated that preconceptional maternal alcohol consumption was associated with lower mean full-scale IQ, overall attention, and sustained attention^2^, which indicated the possible adverse effects of earlier ethanol exposure on the gamete. As maternal alcohol exposure before conception plays an important role in child development, it is biologically plausible that preconceptional paternal alcohol consumption could also have effects on child neurodevelopment through its impacts on sperm^3^.

Spermatogenesis is estimated to take approximately 74 days, which is considered to be a process susceptible to exogenous disturbance^4,5^. The preconceptional period is thus increasingly recognized as a highly sensitive window^6^. Evidence from animal studies has also shown that preconceptional paternal alcohol consumption can induce genetic and epigenetic alterations in sperm that may increase the risks of adverse neurodevelopment in offspring, including attention deficit hyperactivity disorder-like behaviors, anxiety and depression-like behaviors, and cognitive impairment^7–11^. In humans, previous studies about the effects of paternal alcohol consumption on adverse neurodevelopment of the offspring were mainly focused on fathers with alcohol dependence, which indicated the crucial role of genetic transmission in their associations^12,13^. However, we know far less about the effects of alcohol consumption before conception for general fathers, whose alcohol consumption is much lower than those fathers with alcohol dependence.

The present study thus aimed to examine the associations between paternal alcohol consumption during the last 3 months before conception and the risk of child behavioral problems at 2, 4, and 6 years of age.

## Methods

### Study design and participants

**Figure 1:**
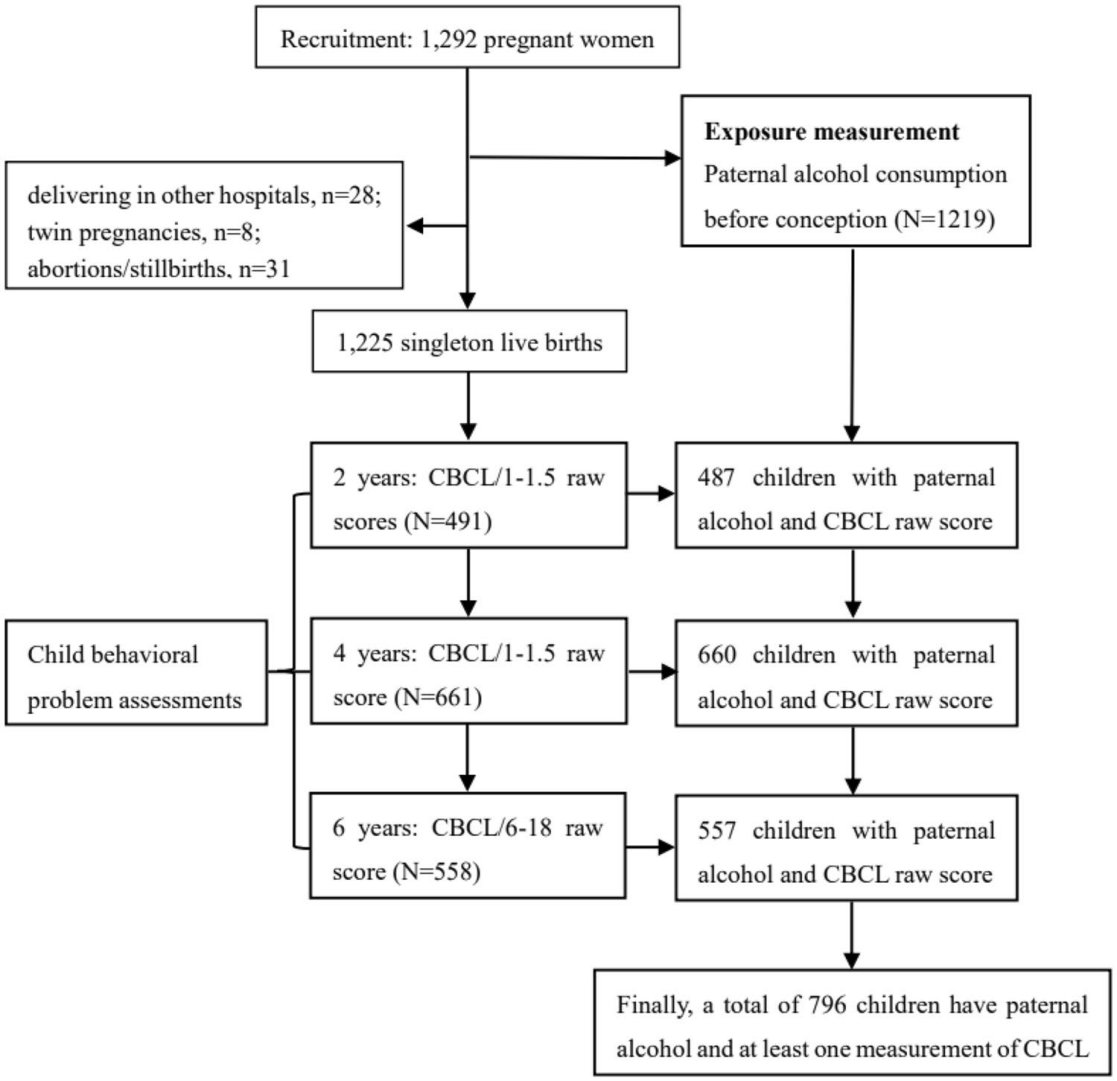
The flow chart depicting the selection procedure of study participants from the S-MBCS study.

The present study was based on the Shanghai-Minhang Birth Cohort Study (S-MBCS), which was designed to determine the effects of early life environmental exposures on the health of offspring^14,15^. From April to December 2012, pregnant women who visited Minhang Maternal and Child Health Hospital for their first prenatal care at 12–16 weeks of gestation were consecutively recruited into this prospective cohort study if they: (1) were native Chinese and residents of Shanghai; (2) had no history of major chronic diseases diagnosed by a physician; (3) intended to deliver their babies in the study hospital; and (4) were willing to take part in scheduled interviews during pregnancy and after delivery. A total of 1,225 singleton mother-infant pairs were recruited in the cohort, among whom 1219 women reported their spouses’ alcohol consumption during the last 3 months before conception at recruitment. The closest caregivers (more than 85% were mothers) completed the inventory of Child Behavior Checklist (CBCL/1.5–5) when their children were 2 and 4 years of age and completed the inventory of CBCL/6–18 when their children were 6 years of age. We restricted the study to 796 children who had information of preconceptional paternal alcohol consumption, and who had at least one CBCL assessment at 2, 4, and 6 years of age, as shown in Fig. 1.

### Ethics

This study involved human participants, and all procedures were performed in accordance with the Declaration of Helsinki. The study protocol was approved by the research ethics committees of the Shanghai Institute of Planned Parenthood Research. Both the pregnant women and their husbands gave informed consent before enrollment in the study and at postnatal follow-up visits for their children’s participation.

### Paternal alcohol consumption before conception

Considering susceptible period of spermatogenesis^4,5^, we defined the time window of exposure as the last 3 months before conception. Paternal alcohol consumption was assessed through questionnaires completed by mothers at 12–16 gestational weeks. Children born to fathers who drank alcohol at least once a week during the last 3 months before conception were classified as exposed, and the rest as unexposed^16,17^. Fathers who drank alcohol were also asked about the type (e.g., beer, liquor, wine, or rice wine) and the average amount of alcohol beverages consumed per week. Alcoholic beverage consumption was recorded in grams as well as in terms of glasses or bottles^16,18^. In line with our previous study, the average amount of alcohol consumption per week from each type of beverage was calculated according to a specific formula: alcohol dose (g/wk) = beverage volume per week (ml) × alcoholic percentage of the beverage (%) × 0.8 g/ml (density of alcohol)^16^. The cumulative exposure of alcohol consumption per week was then calculated as the sum of the average amount of alcohol consumption for each type of beverage.

### Child behavioral problems assessment

Child behavioral problems were assessed using the CBCL when children were 2, 4, and 6 years of age. The CBCL is a psychometric instrument applied widely in clinical and research settings to assess child behavioral problems. The Chinese version of the CBCL/1.5–5 and CBCL/6–18 have been validated for use among preschool (1.5–5 years) and school-aged (6–18 years) children with high reliability and stability^19,20^. The instrument instructs the mother to rate her child's behavior using a 3-category scale ranging from not true to often true, with 0 representing not true, 1 representing somewhat or sometimes true, and 2 representing very true or often true. Higher scores indicating greater behavioral problems. The CBCL/1.5–5, consisting of 99 items, provides measurements on 7 subscales obtained by summing the responses for each item within the subscale, including emotionally reactive, anxious/depressed, somatic complaints, withdrawn, sleep problems, attention problems, and aggressive behavior. Two aggregated scales measuring internalizing and externalizing behavioral problems are generated on the basis of the 7 subscales: internalizing behavior scores are derived from the sum of the emotionally reactive, anxious/depressed, somatic complaints, and withdrawn; externalizing behavior scores are derived from the sum of attention problems and aggressive behavior. In the CBCL 6–18, the 113-item scale is also subdivided into several subscales, namely anxious/depressed, withdrawn/depressed, somatic complaints, social problems, thought problems, attention problems, rule-breaking behavior, and aggressive behavior^21^. Scores on internalizing and externalizing behavior problems of CBCL 6–18, can also be obtained. The Internalizing domain here subsumes three syndrome scales: anxious/depressed, withdrawn/depressed, and somatic complaints. The externalizing domain includes the rule-breaking behavior and aggressive behavior syndrome scales. In the present study, mother–child pairs were followed up by home visits when children were 4, and 6 years of age. At ages 2, the information of CBCL was administered over the mail, as no in- person visits were conducted.

The instrument also provides T scores, which are standardized to a normative population, however, because T scores are truncated, these scores do not allow researchers to differentiate among children with low scores, which are indicative of fewer problems^22^. Thus, we included the CBCL raw scores in the final analyses.

### Other variables

Information was obtained on maternal and paternal demographic characteristics, health condition, medical and reproductive history, and lifestyle factors, such as smoking and drinking habits, at recruitment by a structured questionnaire. Paternal smoking was defined as smoking at least 1 cigarette per week. Passive smoking was defined as indoor inhalation for at least 15 min a day from other smokers^16^. The depressive symptoms of pregnant women were also assessed at recruitment using the Centre for Epidemiological Studies Depression Scale. Information on children’s birth date, gender, and gestational age at birth was extracted from medical records.

### Statistical analyses

#### Main analyses on the associations between preconceptional paternal alcohol consumption and CBCL raw scores

The baseline characteristics of children in exposed and unexposed groups were tabulated using counts and percentages for categorical variables and means (standard deviation, SD) for continuous variables. We also described the distributions of the demographic characteristics of the included and excluded children. We considered the CBCL-scores as count data according to its distribution (Supplementary Table S1). To account for greater variability (variance larger than mean) in the data and the better relative fit than would be expected based on a Poisson model (Supplementary Table S1), negative binomial regression was used to estimate the unadjusted and adjusted rate ratios (risks) (RR) and 95% confidence interval (CI) for the association between paternal alcohol consumption and risk of CBCL raw scores in offspring. In preliminary analyses, we included a multiplicative interaction term (exposure × sex) in statistical models. Potential sex-specific effects seemed to be suggested, thus, we also presented the results of stratified analysis by sex of children.

We also explored the dose–response relationship between preconceptional paternal alcohol consumption and CBCL raw scores by sex of children. Fathers who drank alcohol were categorized by tertiles (lowest: 0–30.72 g/week; middle: 30.72–68.48 g/week; and highest tertiles: > 68.48 g/week) according to the amount of cumulative consumption per week, which lead to 4 categories of exposure: non-drinkers (unexposed group), low (0–30.72 g/week), moderate (30.72–68.48 g/week), and highest (> 68.48 g/week) drinking. Trends tests were assessed by entering the 4 categories of the amount of paternal alcohol consumption as ordinal integer values (i.e., 1–4).

Covariates were examined as potential confounders on the basis of prior knowledge on alcohol-mental health relationship^23^. They were also retained if they caused a ≥ 10% change in the effect estimates for the exposure–outcome associations. Paternal age (years), paternal body-mass index (BMI) (< 18.5, 18.5–24 or > 24.0, kg/m^2^), paternal education (high school or below, college or university, postgraduate or above), paternal smoking before conception (yes or no), gestational age (weeks), sex of offspring, maternal age at conception (years), maternal parity (primiparous, multiparous), maternal depressive symptoms (yes or no, with the cut-off point at 16)^24^, maternal folic acid intake before conception (yes or no), and maternal multivitamin intake during pregnancy (yes or no) were included in the negative binomial regression models.

### Sensitivity analyses

To further rule out the potential confounding effects of paternal smoking, we restricted the analyses to children whose fathers had never smoked before conception. We also re-ran the analysis among children whose mothers were not exposed to passive smoking during pregnancy to examine the robustness of the results. We did not include maternal smoking and maternal drinking in our analyses because few women were active smokers (2.0%) in Shanghai^25^, and only nine women (1.4%) reported alcohol consumption during pregnancy (Supplementary Table S2). Finally, we repeated the analyses in parents who received college or university education, or had normal pre-pregnancy BMI (18.5 < BMI < 24 kg/m^2^) to reduce any concern about the effects of these variables on the results.

All analyses were conducted with SAS 9.4 (SAS Institute Inc, Cary, NC, USA). Two-sided *P*-values of < 0.05 were regarded as statistically significant.

## Results

### Participant characteristics

The demographic characteristics of participants are shown in Table 1. In the present study, a total of 253 (31.8%) children were born to fathers who had alcohol consumption during the last 3 months before conception. Compared with the unexposed group, fathers in the exposed group were more likely to receive lower levels of education, and to smoke before conception. Mothers in the exposed group were more likely to report depressive symptoms during pregnancy or to be exposed to passive smoking, and less likely to take folic acid supplements before conception. Compared to the excluded children of the S-MBCS cohort, mothers of included children were older, better educated, and less likely to be depressed during pregnancy. Parental education of included children was a little higher than that of those excluded. No significant differences in other background characteristics were found (Supplementary Table S2).

**Table 1:** Characteristics of children with and without preconceptional paternal alcohol consumption.

Characteristics	Preconceptional paternal alcohol consumption		
	Exposed (N = 253)	Unexposed (N = 543)	*P*-value^a^
**Paternal characteristics**			
Paternal Age, mean ± SD, years	31.05 (4.65)	30.92 (4.38)	0.69
Paternal body mass index (kg/m^2^)			
< 18.5	11 (4.49)	16 (3.09)	0.58
18.5–24	144 (58.78)	301 (58.22)	
≥ 24	90 (36.73)	200 (38.68)	
Paternal education			
High school or below	68 (26.88)	93 (17.13)	< 0.01
College or university	156 (61.66)	364 (67.03)	
Postgraduate or above	29 (11.46)	86 (15.84)	
Smoking			
Yes	135 (53.57)	125 (23.02)	< 0.0001
No	117 (46.43)	418 (76.98)	
**Maternal characteristics**			
Maternal age(years)	28.68 (3.35)	28.43 (3.41)	0.34
Maternal pre-pregnancy body mass index (kg/m^2^)			
< 18.5	47 (18.65)	97 (18.23)	0.99
18.5–24	184 (73.02)	390 (73.31)	
≥ 24	21 (8.33)	45 (8.46)	
Maternal education			
High school or below	54 (21.34)	112 (20.66)	0.90
College or university	175 (69.17)	373 (68.82)	
Postgraduate or above	24 (9.49)	57 (10.52)	
Family income capia (RMB/per month)			
< 4000	51 (20.4)	106 (19.74)	0.97
4000–7999	99 (39.6)	217 (40.41)	
≥ 8000	100 (40)	214 (39.85)	
Parity			
Primiparous	213 (84.86)	466 (86.46)	0.55
Multiparious	38 (15.14)	73 (13.54)	
Maternal passive smoking before conception			
Yes	149 (58.89)	177 (32.78)	< 0.001
No	104 (41.11)	363 (67.22)	
Depressive symptoms during pregnancy			
Yes	59 (23.32)	83 (15.29)	< 0.01
No	194 (76.68)	460 (84.71)	
Preconceptional folic acid supplement			
Yes	93 (36.90)	263 (48.88)	< 0.01
No	159 (63.10)	275 (51.12)	
**Children’s characteristics**			
Gestational age(weeks)	39.51 (1.44)	39.54 (1.23)	0.74
Sex			
Boy	150 (59.29)	291 (53.89)	0.15
Girl	103 (40.71)	249 (46.11)	

Missing data: Exposed: paternal age (N = 2), paternal body mass index (N = 8), paternal smoking (N = 1), maternal pre-pregnancy body mass index (N = 1), parity (N = 2), preconceptional maternal folic acid supplements (N = 1), family income capita (N = 3), and gestational age (N = 1).

Unexposed: paternal age (N = 2), paternal body mass index (N = 26), maternal pre-pregnancy body mass index (N = 11), maternal education (N = 1), parity (N = 4), maternal passive smoking before conception (N = 3), preconceptional maternal folic acid supplements (N = 5), and family income capita (N = 6).

^a^Two-sample *t* test if continuous and Pearson *χ*^2^ if categorical.

### Associations between paternal alcohol consumption and CBCL raw scores among children at age 2, 4, and 6 years

We found positive patterns (worse neurodevelopment) of paternal alcohol consumption with anxious/depressed, aggressive behavior, and internalizing problem scores in children at 2 years of age, but the associations became attenuated after adjusting for potential confounders.

At age of 4, we found elevated risks of rating scores on emotion reactive, anxious/depressed, sleep problems, and internalizing problems in children born to fathers with alcohol consumption before conception (RR range 1.16–1.23). When we stratified the analyses by sex, the risk of rating scores on anxious/depressed was increased by 33% (RR 1.33, 95% CI 1.09, 1.61) and the risk of rating scores on sleep problems was increased by 25% (RR 1.25, 95% CI 1.00, 1.55) among girls in the exposed group. Boys in the exposed group tend to have higher rating scores on somatic complaints, sleep problems, and internalizing problems. At age 6, paternal alcohol consumption was associated with increased risks of anxious/depressed scores in girls (RR 1.37, 95% CI 1.02, 1.84), and somatic complaints scores in boys (RR 1.65, 95% CI 1.14, 2.38), which was consistent with the findings at age 4. We also found that the risks of thought problem scores were increased by 32% (RR 1.32, 95% CI 1.01, 1.73) among girls in exposed group at age 6. The risks of rule-breaking behaviors and externalizing behaviors scores were increased by 35% (RR 1.35, 95% CI 1.09, 1.67) and 24% (RR 1.24, 95% CI 1.03,1.51), respectively, among boys in the exposed group at age 6, as shown in Table 2.

**Table 2:** Associations between preconceptional paternal alcohol consumption (yes/no) and CBCL raw scores for children at 2, 4 and 6 years of age.

CBCL raw scores	All	All	Boys	Girls
	Crude RR (95% CI)	Adjusted RR (95% CI)^b^	Adjusted RR (95% CI)^c^	Adjusted RR (95% CI)^c^
Children at 2 years of age	N = 484		N = 267	N = 217
Emotionally reactive	1.13 (0.97, 1.32)	1.13 (0.95, 1.34)	1.19 (0.93, 1.53)	1.04 (0.80, 1.34)
Anxious/depressed	1.12 (0.98, 1.28)^#^	1.10 (0.95, 1.27)	1.10 (0.89, 1.35)	1.14 (0.92, 1.40)
Somatic complaints	1.08 (0.95, 1.22)	1.02 (0.89, 1.17)	1.00 (0.82, 1.22)	1.08 (0.88, 1.32)
Withdrawn	1.04 (0.86, 1.27)	1.02 (0.82, 1.26)	1.05 (0.78, 1.41)	0.99 (0.72, 1.36)
Sleep problems	1.06 (0.91, 1.23)	1.07 (0.91, 1.27)	1.16 (0.92, 1.46)	1.04 (0.82, 1.32)
Attention problems	0.91 (0.78, 1.06)	0.87 (0.73, 1.03)	0.82 (0.65, 1.04)	0.95 (0.74, 1.23)
Aggressive behaviors	1.12 (1.00, 1.26)^#^	1.07 (0.94, 1.21)	1.10 (0.92, 1.32)	1.03 (0.84, 1.26)
Internalizing behaviors	1.10 (0.99, 1.22)^#^	1.07 (0.95, 1.21)	1.09 (0.92, 1.29)	1.08 (0.90, 1.28)
Externalizing behaviors	1.09 (0.97, 1.22)	1.04 (0.92, 1.18)	1.05 (0.89, 1.25)	1.02 (0.84, 1.24)

Children at 4 years of age	N = 660		N = 374	N = 286
Emotionally reactive	1.19 (1.03, 1.37)*	1.19 (1.02, 1.39)*	1.18 (0.96, 1.46)	1.17 (0.92, 1.48)
Anxious/depressed	1.19 (1.05, 1.35)*	1.23 (1.07, 1.41)*	1.15 (0.95, 1.39)	1.33 (1.09, 1.61)*
Somatic complaints	1.13 (1.01, 1.28)*	1.12 (0.98, 1.27)^#^	1.18 (1.00, 1.40)^#^	1.03 (0.84, 1.26)
Withdrawn	1.09 (0.91, 1.29)	1.03 (0.85, 1.25)	1.04 (0.80, 1.35)	1.09 (0.82, 1.46)
Sleep problems	1.14 (0.99, 1.31)^#^	1.21 (1.05, 1.40)*	1.20 (0.98, 1.46)^#^	1.25 (1.0023, 1.55)*
Attention problems	1.05 (0.92, 1.20)	1.01 (0.87, 1.16)	1.03 (0.85, 1.24)	0.97 (0.78, 1.22)
Aggressive behaviors	1.12 (1.00, 1.26)^#^	1.07 (0.94, 1.22)	1.15 (0.97, 1.37)	0.99 (0.81, 1.21)
Internalizing behaviors	1.16 (1.04, 1.29)*	1.16 (1.03, 1.30)*	1.16 (0.99, 1.36)^#^	1.16 (0.97, 1.38)
Externalizing behaviors	1.11 (0.99, 1.23)^#^	1.06 (0.94, 1.19)	1.13 (0.96, 1.32)	0.99 (0.82, 1.19)

Children at 6 years of age	N = 557		N = 323	N = 234
Anxious/depressed	1.21 (1.00, 1.46)*	1.28 (1.04, 1.56)*	1.13 (0.86, 1.50)	1.37 (1.02, 1.84)*
Withdrawn/depressed	0.95 (0.79, 1.14)	0.90 (0.74, 1.10)	0.83 (0.63, 1.09)	0.98 (0.73, 1.32)
Somatic complaints ^a^	1.38 (1.07, 1.77)*	1.43 (1.09, 1.88)*	1.65 (1.14, 2.38)*	1.21 (0.79, 1.83)
Social problems	1.18 (1.01, 1.37)*	1.13 (0.96, 1.34)	1.13 (0.90, 1.40)	1.09 (0.85, 1.39)
Thought problems	1.24 (1.05, 1.45)*	1.28 (1.07, 1.53)*	1.23 (0.97, 1.55)^#^	1.32 (1.01, 1.73)*
Attention problems^a^	1.15 (1.01, 1.31)*	1.09 (0.95, 1.25)	1.18 (0.98, 1.43)	0.96 (0.78, 1.18)
Rule-breaking behaviors	1.23 (1.06, 1.43)*	1.24 (1.05, 1.46)*	1.35 (1.09, 1.67)*	1.03 (0.77, 1.36)
Aggressive behaviors ^a^	1.12 (0.97, 1.30)	1.08 (0.92, 1.27)	1.20 (0.98, 1.48)^#^	0.89 (0.68, 1.17)
Internalizing behaviors	1.14 (0.98, 1.33)^#^	1.16 (0.98, 1.37)^#^	1.08 (0.87, 1.34)	1.22 (0.94, 1.58)
Externalizing behaviors	1.16 (1.01, 1.34)*	1.14 (0.98, 1.32)^#^	1.24 (1.03, 1.51)*	0.95 (0.74, 1.22)

*CBCL* Child Behavior Checklist.

*Statistically significant differences (*P*-value < 0.05).

^#^*P-*value < 0.10.

^a^*P*-values of interaction item (preconceptional paternal alcohol consumption*infant sex) < 0.1

^b^Adjusted for paternal age, paternal body mass index, paternal education, paternal smoking, maternal age, parity, maternal depressive symptoms during pregnancy, maternal preconception folic acid supplements, multivitamin supplements during pregnancy, gestational weeks, and sex.

^c^Adjusted for all potential confounding variables above except sex.

Table 3 presents the dose–response relationship between preconceptional paternal alcohol consumption and the CBCL raw scores in the children. The risks of CBCL scores on anxious/depressed and sleep problems in girls at age 4, as well as the risks of somatic complaints and rule-breaking behaviors in boys at age 6 increased with the level of exposure to paternal alcohol consumption (Supplementary Table S3): compared with girls in unexposed group, the risks of anxious/depressed and sleep problems were increased by 47% (RR 1.47, 95% CI 1.07, 2.02) and 46% (RR 1.46, 95% CI 1.02, 2.08), respectively, for girls in the highest drinking group; we also found elevated risks of somatic complaints in boys born to fathers with low drinking (RR 1.85, 95% CI 1.07, 3.19) and highest drinking (RR 2.18, 95% CI 1.31, 3.63), and elevated risks of rule-breaking behaviors (RR 1.42, 95% CI 1.04, 1.92) in boys born to fathers with moderate drinking. Some low-dose effects of paternal alcohol consumption on CBCL raw scores on CBCL scores were also indicated, like the increased risk of somatic complaints, sleep problems in boys at 4 years of age and anxious/depressed and thought problems in girls at 6 years of age. We did not observe patterns of dose–response relationship between preconceptional paternal alcohol consumption and the CBCL raw scores in the children at 2 years of age (Supplementary Table S4).

**Table 3:** Associations between preconceptional paternal alcohol consumption (cumulative consumption per week) and CBCL raw scores for children at 4 and 6 years of age**.**

CBCL raw scores	Boys				Girls			
	Unexposed group	Low drinking (0–30.72 g/week)	Moderate drinking (30.72–68.48 g/week)	Highest drinking (68.48–420.80 g/week)	Unexposed group	Low drinking (0–30.72 g/week)	Moderate drinking (30.72–68.48 g/week)	Highest drinking (68.48–420.80 g/week)
Children at 4 years of age	N = 241	N = 33	N = 43	N = 35	N = 206	N = 26	N = 17	N = 24
Emotionally reactive	1 (ref)	1.36 (0.97, 1.91)	1.02 (0.74, 1.40)	1.25 (0.90, 1.74)	1 (ref)	1.07 (0.75, 1.54)	1.23 (0.80, 1.90)	1.25 (0.85, 1.85)
Anxious/depressed	1 (ref)	1.23 (0.90, 1.68)	1.15 (0.87, 1.51)	1.06 (0.78, 1.44)	1 (ref)	1.22 (0.91, 1.64)	1.40 (0.98, 1.98)	1.47 (1.07, 2.02)*
Somatic complaints	1 (ref)	1.42 (1.09, 1.84)*	1.20 (0.94, 1.54)	1.08 (0.82, 1.42)	1 (ref)	0.92 (0.67, 1.26)	1.16 (0.82, 1.65)	1.02 (0.73, 1.42)
Withdrawn	1 (ref)	1.17 (0.76, 1.78)	0.87 (0.59, 1.30)	0.96 (0.63, 1.46)	1 (ref)	0.82 (0.52, 1.29)	1.21 (0.72, 2.02)	1.28 (0.80, 2.03)
Sleep problems	1 (ref)	1.70 (1.26, 2.30)*	0.96 (0.71, 1.30)	0.93 (0.67, 1.30)	1 (ref)	1.11 (0.80, 1.53)	1.27 (0.85, 1.90)	1.46 (1.02, 2.08)*
Attention problems	1 (ref)	1.02 (0.75, 1.39)	1.03 (0.78, 1.36)	1.07 (0.80, 1.44)	1 (ref)	0.81 (0.56, 1.17)	0.82 (0.52, 1.27)	1.31 (0.93, 1.83)
Aggressive behaviors	1 (ref)	1.30 (0.98, 1.71)	1.22 (0.95, 1.56)	0.98 (0.74, 1.28)	1 (ref)	0.88 (0.64, 1.20)	1.05 (0.72, 1.53)	1.14 (0.82, 1.59)
Internalizing behaviors	1 (ref)	1.33 (1.03, 1.72)*	1.09 (0.87, 1.38)	1.10 (0.86, 1.41)	1 (ref)	1.03 (0.79, 1.35)	1.24 (0.90, 1.71)	1.25 (0.93, 1.67)
Externalizing behaviors	1 (ref)	1.25 (0.96, 1.61)	1.17 (0.92, 1.47)	1.00 (0.78, 1.29)	1 (ref)	0.86 (0.65, 1.15)	1.01 (0.72, 1.42)	1.17 (0.87, 1.59)

Children at 6 years of age	N = 217	N = 27	N = 35	N = 31	N = 169	N = 20	N = 15	N = 19
Anxious/depressed	1 (ref)	1.20 (0.76, 1.90)	1.16 (0.77, 1.77)	1.18 (0.76, 1.83)	1 (ref)	1.90 (1.27, 2.86)*	0.87 (0.50, 1.52)	1.39 (0.86, 2.26)
Withdrawn/depressed	1 (ref)	0.72 (0.44, 1.16)	0.89 (0.59, 1.34)	0.70 (0.45, 1.09)	1 (ref)	1.17 (0.76, 1.80)	0.74 (0.42, 1.31)	0.98 (0.60, 1.59)
Somatic complaints	1 (ref)	1.85 (1.07, 3.19)*	1.44 (0.83, 2.48)	2.18 (1.31, 3.63)*	1 (ref)	1.78 (1.03, 3.09)*	0.76 (0.34, 1.68)	1.10 (0.53, 2.26)
Social problems	1 (ref)	1.20 (0.84, 1.72)	1.18 (0.85, 1.64)	1.12 (0.80, 1.58)	1 (ref)	1.52 (1.09, 2.14)*	0.89 (0.57, 1.38)	0.85 (0.55, 1.30)
Thought problems	1 (ref)	1.53 (1.06, 2.20)*	0.99 (0.69, 1.42)	1.27 (0.88, 1.84)	1 (ref)	1.76 (1.22, 2.52)*	0.77 (0.46, 1.31)	1.44 (0.92, 2.24)
Attention problems	1 (ref)	1.27 (0.94, 1.72)	1.06 (0.80, 1.41)	1.21 (0.90, 1.62)	1 (ref)	1.10 (0.81, 1.49)	0.72 (0.49, 1.06)	1.04 (0.74, 1.46)
Rule-breaking behaviors	1 (ref)	1.35 (0.96, 1.90)	1.42 (1.04, 1.92)*	1.27 (0.91, 1.77)	1 (ref)	1.09 (0.71, 1.68)	0.68 (0.39, 1.19)	0.83 (0.50, 1.38)
Aggressive behaviors	1 (ref)	1.37 (0.98, 1.92)	1.14 (0.84, 1.56)	1.00 (0.72, 1.39)	1 (ref)	1.03 (0.68, 1.56)	0.66 (0.40, 1.09)	0.86 (0.54, 1.38)
Internalizing behaviors	1 (ref)	1.09 (0.76, 1.56)	1.08 (0.78, 1.51)	1.12 (0.80, 1.57)	1 (ref)	1.63 (1.12, 2.36)*	0.82 (0.52, 1.32)	1.21 (0.78, 1.87)
Externalizing behaviors	1 (ref)	1.36 (0.99, 1.87)	1.22 (0.91, 1.64)	1.08 (0.80, 1.47)	1 (ref)	1.07 (0.73, 1.56)	0.65 (0.41, 1.04)	0.89 (0.57, 1.37)

Adjusted for paternal age, paternal body mass index, paternal education, paternal smoking, maternal age, parity, maternal depressive symptoms during pregnancy, maternal preconception folic acid supplements, multivitamin supplements during pregnancy, and gestational weeks.

*CBCL* Child Behavior Checklist.

*Statistically significant differences (*P*-value < 0.05).

### Sensitivity analyses

When we repeated the analyses in children without paternal smoking, nearly all the effect sizes were more pronounced, especially for sleep problems of boys and anxious/depressed of girls at 2 years of age (Table 4). Similar patterns were also observed in the children whose mothers were not exposed to passive smoking during pregnancy and received college or university education, whose fathers had normal BMI and received college or university education, although some associations became non-significant mostly due to the reduced sample size (Supplementary Table S5-S8). We also found that the associations of paternal alcohol consumption with anxious/depressed, sleep problems, and thought problems in girls whose mothers had normal pre-pregnancy BMI were attenuated (Supplementary Table S9).

**Table 4:** Associations between preconceptional paternal alcohol consumption and CBCL raw scores at 2, 4 and 6 years of age in children without paternal smoking.

CBCL raw scores	All	Boys	Girls
	Adjusted RR (95% CI)^a^	Adjusted RR (95% CI)^b^	Adjusted RR (95% CI)^b^
Children at 2 years of age	N = 341	N = 196	N = 145
Emotionally reactive	1.19 (0.95, 1.48)	1.16 (0.86, 1.55)	1.19 (0.84, 1.69)
Anxious/depressed	1.16 (0.97, 1.4)	1.11 (0.86, 1.42)	1.28 (0.97, 1.70)^#^
Somatic complaints	1.02 (0.86, 1.22)	0.98 (0.78, 1.23)	1.03 (0.76, 1.38)
Withdrawn	1.17 (0.89, 1.54)	1.06 (0.74, 1.52)	1.22 (0.81, 1.84)
Sleep problems	1.20 (0.98, 1.48)^#^	1.28 (0.98, 1.68)^#^	1.24 (0.88, 1.73)
Attention problems	0.92 (0.74, 1.15)	0.82 (0.61, 1.11)	1.12 (0.79, 1.58)
Aggressive behaviors	1.08 (0.92, 1.28)	1.03 (0.83, 1.28)	1.13 (0.86, 1.48)
Internalizing behaviors	1.13 (0.97, 1.32)	1.08 (0.88, 1.32)	1.18 (0.92, 1.51)
Externalizing behaviors	1.06 (0.91, 1.24)	1.00 (0.81, 1.23)	1.13 (0.88, 1.46)

Children at 4 years of age	N = 448	N = 255	N = 193
Emotionally reactive	1.21 (0.99, 1.47)^#^	1.09 (0.84, 1.42)	1.35 (0.99, 1.85)^#^
Anxious/depressed	1.27 (1.07, 1.51)*	1.08 (0.85, 1.38)	1.58 (1.24, 2.00)*
Somatic complaints	1.05 (0.89, 1.24)	1.10 (0.88, 1.36)	0.97 (0.74, 1.27)
Withdrawn	1.01 (0.79, 1.30)	1.00 (0.72, 1.40)	1.15 (0.78, 1.69)
Sleep problems	1.22 (1.01, 1.47)*	1.27 (0.99, 1.63)^#^	1.20 (0.91, 1.60)
Attention problems	1.06 (0.89, 1.26)	1.07 (0.85, 1.35)	1.06 (0.80, 1.41)
Aggressive behaviors	1.06 (0.90, 1.25)	1.10 (0.89, 1.37)	1.02 (0.79, 1.33)
Internalizing behaviors	1.15 (1.00, 1.34)^#^	1.09 (0.90, 1.33)	1.28 (1.02, 1.61)*
Externalizing behaviors	1.07 (0.92, 1.24)	1.10 (0.90, 1.36)	1.02 (0.81, 1.30)

Children at 6 years of age	N = 379	N = 224	N = 155
Anxious/depressed	1.47 (1.14, 1.91)*	1.18 (0.83, 1.70)	1.72 (1.18, 2.50)*
Withdrawn/depressed	0.94 (0.72, 1.23)	0.85 (0.59, 1.23)	0.97 (0.65, 1.44)
Somatic complaints	1.57 (1.11, 2.21)*	1.79 (1.16, 2.76)*	1.21 (0.69, 2.15)
Social problems	1.22 (0.99, 1.50)^#^	1.15 (0.87, 1.51)	1.21 (0.88, 1.67)
Thought problems	1.28 (1.03, 1.59)*	1.17 (0.88, 1.56)	1.37 (0.96, 1.94)^#^
Attention problems	1.19 (0.99, 1.43)^#^	1.28 (1.00, 1.63)^#^	1.01 (0.76, 1.33)
Rule-breaking behaviors	1.28 (1.05, 1.58)*	1.45 (1.13, 1.85)*	0.89 (0.61, 1.32)
Aggressive behaviors	1.23 (1.00, 1.52)*	1.30 (0.99, 1.69)^#^	0.97 (0.69, 1.38)
Internalizing behaviors	1.30 (1.05, 1.61)*	1.15 (0.87, 1.52)	1.37 (0.98, 1.91)^#^
Externalizing behaviors	1.23 (1.02, 1.50)*	1.33 (1.04, 1.71)*	0.95 (0.69, 1.29)

*CBCL* Child Behavior Checklist.

*Statistically significant differences (*P*-value < 0.05).

^#^*P*-value < 0.10.

^a^Adjusted for paternal age, paternal body mass index, paternal education, maternal age, parity, maternal depressive symptoms during pregnancy, maternal preconception folic acid supplements, multivitamin supplements during pregnancy, gestational weeks, and sex.

^b^Adjusted for all potential confounding variables above except sex.

## Discussion

This prospective cohort study provided the first epidemiological evidence implying that children born to fathers who drank alcohol before conception may have higher risks of experiencing behavioral problems. Paternal alcohol consumption before conception was associated with higher rating scores for anxious/depressed in girls and somatic complaints in boys at 4 and 6 years of age. A dose–response relationship was observed on anxious/depressed and sleep problems among girls at 4 years of age, and on somatic complaints and rule-breaking behaviors among boys at 6 years of age.

Animal studies have provided clear evidence that offspring sired by ethanol-exposed males in the absence of maternal ethanol exposure showed more adverse behavioral development^11^, including increased risk of anxiety- and depression-like behaviors^7,10^, increased in activity and sensorimotor integration deficits, as well as decreased balance, coordination, and short-term motor learning^26^. Previous human studies mostly focused on the effects of paternal alcoholism on psychiatric disorders, including increased risk of externalizing symptoms and attention-deficit hyperactivity disorder^12,13,27,28^. However, none of these studies collected information on paternal alcohol consumption before conception. Although epidemiological studies on the potential effects of paternal preconceptional alcohol consumption on neurodevelopment are still limited, the hypothesis of increased risks of development problems of the offspring associated with paternal preconceptional alcohol consumption is not novel. Several human studies have shown that preconceptional paternal alcohol consumption was associated with lower birthweight^29^, microcephaly^30^, fetal birth defects^31^, increased risk of acute lymphoblastic leukemia^32^, and poor reproductive development^16^. Combined with our findings in the present study, these results suggested that preconception period may be a critical window for offspring development.

Several mechanisms could account for the effects of preconceptional paternal alcohol exposure on neurodevelopment in offspring. First, preconceptional alcohol exposure could increase the risk of child behavior problems via epigenetic modifications of sperm. Environmental conditions during the preconception period have been demonstrated to shape sperm epigenetics that can be heritable and influence offspring phenotypes^33,34^. The association between paternal alcohol exposure and behavioral alterations in offspring may be explained by altered genomic imprinting mediated by changes in DNA methylation of specific genes^10^. Additionally, small noncoding RNAs have also been confirmed to play a key role in paternal alcohol consumption^34,35^. Second, another potential mechanism underlying the adverse effects of paternal alcohol consumption on child behavioral problem might be genetic factors. Fathers with alcohol dependence may transmit genetic vulnerability to offspring not only for alcohol exposure but also for behavioral problems^13,27^. Genetic transmission was also supported by many family, twin and adoption studies, which found that strong genetic components are in the link between parental drinking and behavioral control in their offspring^12,13,27^. Additionally, that alcohol might produce alterations in the germ cell line which lead to chromosomal abnormalities or gene mutations in sperm and then transferred to offspring was also suggested^36^.

Studies that have examined the associations between alcohol consumption and adverse health outcomes in adults point towards different dose–response curves: a U-shape association^37^, a J-shape association^38^, inverted-U shape^39^ between consumption patterns and health outcomes were all reported. Although we tried to demonstrate the dose–response relationship in our present study, our ability is limited due to the relatively small sample size in each dose category and lack of subjects with paternal heavy alcohol consumption before conception (1.6%). Nevertheless, statistically significant dose–response relationship of some domains (e.g., anxious/depressed) was found in total population at different ages, although they did not reach significance after sex-stratification largely due to limited statistical power. Future studies with larger sample size are warranted to confirm the dose–response relationship.

The present study has several strengths. One major strength of the present study was the prospective design, which could provide potential causality between preconceptional paternal alcohol consumption and the risk of child behavioral problem and reduce recall bias. Additionally, we collected detailed information on covariates, which ensured careful consideration on a number of variables related to paternal alcohol consumption or outcome of interest in the analyses.

Our findings should also be considered in the context of some limitations. First, it has been reported that hyperactivity and diminished cognitive abilities in children are related to biological fathers with alcohol dependence rather than to adoptive fathers^36,40,41^, which indicates that the observed associations are less likely to be explained by social and child-raising factors. However, it cannot be ruled out that social and child-raising factors specific to fathers who drink alcohol may contribute to the observed associations. Second, information on preconceptional paternal alcohol consumption was reported by the children’s mothers during pregnancy. Although there was a very good inter-rater agreement between the parental reports of spouses’ drinking practices^23^, alcohol consumption by fathers was not evaluated by objective measurements of biomarkers such as gamma-glutamyl transferase and thus the misclassification of exposure may have been introduced. In addition, we only collected information on paternal alcohol consumption during the last 3 months before conception on the basis that spermatogenesis is a susceptible period. However, there may have been fathers who ceased drinking when they planned to conceive and thus whose children were classified as unexposed. Earlier paternal alcohol consumption can also affect the epigenetic milieu of sperm that could be passed on to offspring^11^. Such misclassification would lead to under-estimation of the true association. Third, quantitative measurement of exposure in this study relied on average alcohol consumption per week. Averaging alcohol exposure could obscure the fact that there were important subgroups of fathers who were binge drinkers or who drank more heavily in one period than in the rest^42^. The effects of the pattern of alcohol consumption may need to be evaluated in future studies. Fourth, family-based designs have shown that parental history of alcohol dependence/ abuse has adverse effects on neurodevelopment of the offspring^12,13^. If heavy alcohol use was defined as drinking at least 21 units (one unit corresponds to 8 g of alcohol) per week according to the UK alcohol guidelines^23^, only 13 (1.6%) were categorized as heavy drinking in the present study. Although we did not collect the information about the maternal preconceptional alcohol consumption, it was reported that only a few women drink (4.5%) in China^25,43^. Thus, the results were less likely be affected by the parental history of alcohol dependence/abuse in this study. Additionally, we lack information about paternal psychiatric disorders prior to conception, which has been reported to be associated with the offspring development^44^. Fifth, our study population may under-represent the sample of lower socio-economic status because more than 70% of parents have college degree or above, which may restrict the generalizability of our findings. Lastly, CBCL at ages of 2 years was assessed by mail in our study, and the samples were relatively small, which may contribute to the nonsignificant results in children at 2 years of age. Future studies with larger samples are needed to confirm our findings.

The present study added to the epidemiological evidence that children born to fathers with preconceptional alcohol consumption may have a higher risk of experiencing neurobehavioral problems. Life course epidemiology and Mendelian inheritance have long been grounded in assumptions on the causal inference of maternal exposures occurring during pregnancy and early life^45^. While maternal antenatal risk factors for adverse offspring development remain an important focus for intervention, findings of the present study combined with converging evidence highlight the effects of paternal exposure prior to conception on offspring development. Determining if the paternal preconceptional period represent a window of heightened vulnerability would improve our understanding of modifiable risk factors for children’s health and wellbeing^33^. Besides, our findings calls for greater attention to this issue given that exposure to paternal alcohol consumption is widespread (nearly one-third of fathers consumed alcohol before conception in China^31^, and many pregnancies are unplanned^46^. Importantly, there are no guidelines on safe drinking levels for men in couples trying for a pregnancy^30^. While the present study need to be confirmed by further studies, our findings highlight the risk of paternal alcohol consumption prior to conception as well as the necessity for future research in this field.

## Conclusions

Our study provided very preliminary evidence that paternal alcohol consumption during the last 3 months before conception may increase the risk of child behavioral problems. Although consistent with converging evidence from animal models, replication in birth cohort studies with larger samples is needed.

## Supplementary Information

Supplementary Tables.

## Data Availability

The datasets generated and/or analyzed during the current study are not publicly available due to subject confidentiality but are available from the corresponding author on reasonable request.
