# Supplementary material for: Preconceptional paternal alcohol consumption and the risk of child behavioral problems: a prospective cohort study
Source: Sci Rep. 2022 Jan 27;12:1508. doi: 10.1038/s41598-022-05611-2 (PMC8795263; doi:10.1038/s41598-022-05611-2)
Supplement: Supplementary file 1 — Supplementary Tables. [file 41598_2022_5611_MOESM1_ESM.pdf]

Preconceptional paternal alcohol consumption and the risk of child behavioral problems: a prospective cohort study

Min Luan[1, #], Xiaohua Zhang[2, #], Guanghong Fang[3], Hong Liang[3], Fen Yang[3], Xiuxia Song[3], Yao Chen[3], Wei Yuan[3], Maohua Miao[3, *]

[1] NHC Key Lab. of Reproduction Regulation (Shanghai Institute for Biomedical and Pharmaceutical Technologies), School of Public Health, Fudan University, Shanghai, 200237, China.

[2] Minhang Maternal and Child Health Hospital, Shanghai, 201102, China.

[3] NHC Key Lab. of Reproduction Regulation (Shanghai Institute for Biomedical and Pharmaceutical Technologies), Fudan University, Shanghai, China.

[#] **These authors contributed equally to this manuscript.**

[*]**Corresponding author**

Maohua Miao, NHC Key Lab. of Reproduction Regulation (Shanghai Institute for Biomedical and Pharmaceutical Technologies), Fudan University, 779 Lao Hu Min Road, Shanghai, 200237, China.

E-mail: miaomaohua@live.com;

Contents of supplementary materials

Table S1. Distribution of CBCL raw scores from 2 to 6 years of age.
Table S2. Characteristics of included and excluded children.
Table S3. Trend analysis of associations between preconceptional paternal alcohol consumption (cumulative consumption per week) and CBCL raw scores for children at 2, 4 and 6 years of age.
Table S4. Associations between preconceptional paternal alcohol consumption (cumulative consumption per week) and CBCL raw scores for children at 2 years of age.
Table S5. Associations between preconceptional paternal alcohol consumption and CBCL raw scores at 2, 4 and 6 years of age in children without maternal passive smoking.
Table S6. Associations between preconceptional paternal alcohol consumption (yes/no) and CBCL raw scores for children at 2, 4 and 6 years of age among mothers with education at college or university.
Table S7. Associations between preconceptional paternal alcohol consumption (yes/no) and CBCL raw scores for children at 2, 4 and 6 years of age among fathers with education at college or university.
Table S8. Associations between preconceptional paternal alcohol consumption (yes/no) and CBCL raw scores for children at 2, 4 and 6 years of age among fathers with normal BMI (BMI 18.5~24 kg/m^2).
Table S9. Associations between preconceptional paternal alcohol consumption (yes/no) and CBCL raw scores for children at 2, 4 and 6 years of age among mothers with normal pre-pregnancy BMI (BMI 18.5~24 kg/m^2).

Table S1. Distribution of CBCL raw scores from 2 to 6 years of age.

	All subjects				Boys				Girls			
	Mean	Median	P25	P75	Mean	Median	P25	P75	Mean	Median	P25	P75
**Children at 2 years of age**	N=487				N=267				N=217			
Emotionally reactive	2.35 (1.95)	2	1	3	2.34 (1.97)	2	1	3	2.37 (1.95)	2	1	4
Anxious/depressed	2.57 (1.80)	2	1	4	2.58 (1.83)	2	1	4	2.57 (1.77)	2	1	3
Somatic Complaints	2.51 (1.72)	2	1	3	2.46 (1.68)	2	1	3	2.58 (1.76)	2	1	4
Withdrawn	1.71 (1.80)	1	0	3	1.66 (1.74)	1	0	3	1.77 (1.89)	1	0	3
Sleep problems	2.94 (2.34)	2	1	4	2.90 (2.28)	3	1	4	3 (2.42)	2	1	4
Attention problems	1.61 (1.25)	1	1	2	1.68 (1.24)	1	1	3	1.51 (1.25)	1	1	2
Aggressive behaviors	9.30 (5.45)	9	5	13	9.80 (5.53)	9	5	13	8.71 (5.27)	8	5	12
Internalizing behaviors	9.15 (5.18)	8	5	12	9.04 (5.22)	8	5	12	9.29 (5.15)	9	6	12
Externalizing behaviors	10.9 (6.12)	11	6	15	11.48 (6.22)	11	6	16	10.22 (5.92)	10	6	14
**Children at 4 years of age**	N=660				N=374				N=286			
Emotionally reactive	2.22 (1.97)	2	1	3	2.28 (2.02)	2	1	4	2.14 (1.89)	2	1	3
Anxious/depressed	2.56 (1.99)	2	1	4	2.49 (2.01)	2	1	4	2.64 (1.96)	2	1	4
Somatic Complaints	2.43 (1.83)	2	1	3	2.41 (1.80)	2	1	3	2.46 (1.88)	2	1	3
Withdrawn	1.67 (1.82)	1	0	2	1.68 (1.83)	1	0	2	1.67 (1.82)	1	0	2
Sleep problems	2.48 (2.08)	2	1	4	2.47 (2.13)	2	1	3	2.49 (2.03)	2	1	4
Attention problems	1.69 (1.36)	2	1	3	1.79 (1.45)	2	1	3	1.55 (1.24)	1	1	2
Aggressive behaviors	7.14 (4.85)	6	4	10	7.62 (5.08)	7	4	11	6.50 (4.45)	6	3	9
Internalizing behaviors	8.87 (5.8)	8	5	12	8.84 (5.79)	8	5	12	8.92 (5.81)	8	5	12
Externalizing behaviors	8.81 (5.57)	8	5	12	9.39 (5.88)	9	5	13	8.06 (5.04)	8	4	11
**Children at 6 years of age**	N=557				N=323				N=234			

Anxious/depressed	1.67 (1.81)	1	0	2	1.56 (1.76)	1	0	2	1.82 (1.86)	1	0	3
Withdrawn/depressed	1.18 (1.20)	1	0	2	1.18 (1.23)	1	0	2	1.18 (1.16)	1	0	2
Somatic complaints	0.58 (0.84)	0	0	1	0.51 (0.76)	0	0	1	0.68 (0.94)	0	0	1
Social problems	2.10 (1.75)	2	1	3	2.17 (1.83)	2	1	3	2.01 (1.63)	2	1	3
Thought problems	1.96 (1.80)	2	1	3	2.07 (1.89)	2	1	3	1.82 (1.65)	1.5	1	3
Attention problems	3.63 (2.54)	3	2	5	3.93 (2.77)	4	2	6	3.22 (2.13)	3	2	4
Rule-breaking behaviors	1.74 (1.52)	1	1	3	1.94 (1.62)	2	1	3	1.46 (1.33)	1	0	2
Aggressive behaviors	4.16 (3.41)	3	2	6	4.51 (3.58)	3	2	7	3.66 (3.09)	3	1	5
Internalizing behaviors	3.43 (2.95)	3	1	5	3.25 (2.79)	3	1	4	3.69 (3.13)	3	1	5
Externalizing behaviors	5.87 (4.52)	5	2	8	6.44 (4.83)	5	3	9	5.07 (3.92)	4	2	7

Table S2. Characteristics of included and excluded children.

	Excluded	Included	t/χ^2	P-value
	Mean (SD) / n	Mean (SD) / n		
**Paternal Characteristics**				
Paternal age (years)	30.9 (4.43)	31.0 (4.46)	-0.27	0.79
Preceptional paternal alcohol consumption				
Yes	140 (33.1)	253 (31.8)	0.22	0.64
No	283 (66.9)	543 (68.2)		
Preceptional paternal alcohol consumption				
0	283 (66.9)	543 (68.2)	2.01	0.73
Low drinking (0-30.72 g/wk)	39 (9.2)	77 (9.7)		
Moderate drinking (30.72-68.48 g/wk)	40 (9.5)	66 (8.3)		
High drinking (>68.48 g/week)	35 (8.3)	73 (9.2)		
Missing data	26 (6.2)	37 (4.7)		
Paternal body mass index(kg/m^2)				
< 18.5	15 (3.6)	27 (3.4)	2.69	0.44
18.5 - 24	256 (60.5)	445 (55.9)		
≥24	137 (32.3)	290 (39.4)		
Missing data	15 (3.6)	34 (4.3)		
Paternal education*				
High school or below	118 (27.9)	161 (20.2)	22.53	<0.001
College or university	278 (65.7)	520 (65.3)		
Postgraduate or above	27 (6.4)	115 (14.5)		
Smoking				
Yes	155 (36.6)	260 (32.7)	2.44	0.30
No	268 (63.4)	535 (67.2)		
Missing data	0 (0.0)	1 (0.1)		
**Maternal Characteristics**				
Maternal age (years)*	28.0 (3.4)	28.5 (3.4)	-2.31	0.02
Maternal body mass index(kg/m^2)				
< 18.5	99 (23.4)	144 (18.1)	5.78	0.12
18.5 - 24	283 (66.9)	574 (72.1)		
≥24	32 (7.6)	66 (8.3)		
Missing data	9 (2.1)	12 (1.5)		
Maternal education*				
High school or lower	125 (29.6)	166 (20.9)	21.22	<0.001
College or university	279 (66.0)	548 (68.9)		
Postgraduate or above	18 (4.3)	81(10.2)		
Missing data	1 (0.2)	1 (0.1)		
Family income capita (RMB/per month)				
<4000,	95 (22.5)	157 (19.7)	2.47	0.48
4000-7999	169 (40.0)	316 (39.7)		
≥8000	152 (35.9)	314 (39.5)		
Missing data	7 (1.7)	9 (1.1)		

Parity				
Primiparous	345 (81.6)	679 (85.3)	3.06	0.22
Multiparious	75 (17.7)	111 (13.9)		
Missing data	3 (0.7)	6 (0.8)		
Maternal drinking during pregnancy				
Yes	2 (0.7)	9 (1.1)	18.90	<0.001
No	305 (72.1)	651 (81.8)		
Missing data	116 (27.4)	136 (17.1)		
Maternal passive smoking before conception				
Yes	168 (39.7)	326 (41.0)	0.36	0.84
No	254 (60.1)	467 (58.7)		
Missing data	1 (0.2)	3 (0.4)		
Maternal passive smoking during pregnancy				
Yes	127(40.58)	277(41.97)	13.81	0.001
No	186(59.42)	383(58.03)		
Missing data	110 (26.0)	136 (17.1)		
Depressive symptoms during pregnancy				
Yes	98 (23.2)	142 (17.8)	4.96	0.03
No	325 (76.8)	654 (82.2)		
Preconceptional folic acid supplement				
Yes	181 (42.8)	356 (44.7)	0.81	0.67
No	240 (56.7)	434 (54.5)		
Missing data	2 (0.5)	6 (0.8)		
**Children's Characteristics**				
Gestational age(weeks)	39.4 (1.5)	39.5 (1.3)	-1.41	0.16
Sex				
Boy	222 (52.5)	441 (55.4)	2.69	0.26
Girl	201 (47.5)	352 (44.2)		
Missing data	0 (0.0)	3 (0.4)		

* Statistically significant differences (p-value < 0.05).

Table S3. Trend analysis of associations between preconceptional paternal alcohol consumption (cumulative consumption per week) and CBCL raw scores for children at 2, 4 and 6 years of age.

CBCL Raw Scores	All[a]		Boys[b]		Girls[b]	
	RR (95% CI)	*P*-trend	RR (95% CI)	*P*-trend	RR (95% CI)	*P*-trend
**Children at 2 years of age**	N=463		N=254		N=206	
Emotionally reactive	1.04 (0.96, 1.13)	0.34	1.08 (0.96, 1.22)	0.19	0.99 (0.87, 1.12)	0.82
Anxious/depressed	1.04 (0.97, 1.11)	0.31	1.05 (0.95, 1.16)	0.34	1.04 (0.94, 1.15)	0.49
Somatic complaints	1.03 (0.96, 1.10)	0.38	1.05 (0.96, 1.15)	0.27	1.02 (0.92, 1.12)	0.72
Withdrawn	0.95 (0.85, 1.05)	0.30	0.95 (0.82, 1.09)	0.45	0.96 (0.82, 1.12)	0.59
Sleep problems	1.04 (0.96, 1.12)	0.39	1.06 (0.95, 1.19)	0.28	1.03 (0.92, 1.15)	0.64
Attention problems	0.96 (0.88, 1.04)	0.29	0.95 (0.85, 1.07)	0.41	0.97 (0.86, 1.09)	0.60
Aggressive behaviors	1.03 (0.97, 1.10)	0.30	1.06 (0.97, 1.16)	0.17	1 (0.9, 1.1)	0.94
Internalizing behaviors	1.02 (0.96, 1.08)	0.49	1.05 (0.96, 1.13)	0.29	1 (0.92, 1.09)	0.96
Externalizing behaviors	1.02 (0.96, 1.09)	0.45	1.05 (0.96, 1.14)	0.29	0.99 (0.91, 1.09)	0.89
**Children at 4 years of age**	N=625		N=352		N=273	
Emotionally reactive	1.08 (1.00, 1.16)	0.05	1.06 (0.96, 1.18)	0.23	1.08 (0.97, 1.22)	0.17
Anxious/depressed	1.09 (1.02, 1.17)	0.01	1.04 (0.95, 1.14)	0.43	1.15 (1.05, 1.27)	**<0.01**
Somatic complaints	1.04 (0.97, 1.10)	0.25	1.05 (0.97, 1.14)	0.22	1.02 (0.92, 1.12)	0.75
Withdrawn	1.00 (0.92, 1.10)	0.93	0.97 (0.86, 1.11)	0.69	1.07 (0.93, 1.23)	0.34
Sleep problems	1.05 (0.98, 1.13)	0.16	1.00 (0.90, 1.10)	0.93	1.13 (1.02, 1.26)	**0.02**
Attention problems	1.03 (0.96, 1.10)	0.41	1.02 (0.93, 1.12)	0.65	1.04 (0.93, 1.16)	0.48
Aggressive behaviors	1.03 (0.97, 1.09)	0.38	1.03 (0.95, 1.12)	0.48	1.03 (0.93, 1.14)	0.56
Internalizing behaviors	1.06 (1.00, 1.12)	0.04	1.04 (0.97, 1.12)	0.29	1.08 (0.99, 1.18)	0.07
Externalizing behaviors	1.03 (0.97, 1.09)	0.36	1.03 (0.95, 1.11)	0.49	1.03 (0.94, 1.13)	0.50
**Children at 6 years of age**	N=533		N=310		N=223	
Anxious/depressed	1.11 (1.01, 1.22)	**0.04**	1.06 (0.93, 1.22)	0.36	1.12 (0.97, 1.30)	0.13

Withdrawn/depressed	0.94 (0.85, 1.04)	0.21	0.90 (0.79, 1.03)	0.11	0.97 (0.84, 1.13)	0.70
Somatic complaints	1.17 (1.03, 1.33)	0.02	1.28 (1.08, 1.5)	**<0.01**	1.03 (0.84, 1.27)	0.75
Social problems	1.04 (0.96, 1.13)	0.31	1.05 (0.95, 1.17)	0.31	0.98 (0.86, 1.11)	0.75
Thought problems	1.09 (1.00, 1.19)	**0.04**	1.07 (0.96, 1.2)	0.23	1.11 (0.97, 1.27)	0.13
Attention problems	1.03 (0.96, 1.10)	0.38	1.06 (0.97, 1.16)	0.20	0.98 (0.88, 1.08)	0.68
Rule-breaking behaviors	1.07 (0.98, 1.16)	0.13	1.12 (1.01, 1.23)	**0.03**	0.92 (0.79, 1.07)	0.29
Aggressive behaviors	1.00 (0.92, 1.08)	0.95	1.03 (0.93, 1.13)	0.59	0.92 (0.81, 1.06)	0.27
Internalizing behaviors	1.06 (0.98, 1.15)	0.13	1.04 (0.94, 1.15)	0.46	1.07 (0.93, 1.21)	0.34
Externalizing behaviors	1.02 (0.95, 1.10)	0.56	1.06 (0.96, 1.16)	0.26	0.93 (0.82, 1.06)	0.29

Abbreviations: CBCL, Child Behavior Checklist

[a] Adjusted for paternal age, paternal body mass index, paternal education, paternal smoking, maternal age, parity, maternal depressive symptoms during pregnancy, maternal preconception folic acid supplements, multivitamin supplements during pregnancy, gestational weeks, and sex.

[b] Adjusted for all potential confounding variables above except sex in stratified-sex analyses.

Table S4. Associations between preconceptional paternal alcohol consumption (cumulative consumption per week) and CBCL raw scores for children at 2 years of age**.**

CBCL Raw Scores	Boys				Girls			
	Unexposed group	Low drinking (0-30.72 g/wk)	Moderate drinking (30.72-68.48 g/wk)	High drinking (68.48-420.80 g/wk)	Unexposed group	Low drinking (0-30.72 g/wk)	Moderate drinking (30.72-68.48 g/wk)	High drinking (68.48-420.80 g/wk)
**Children at 2 years of age**	N=174	N=24	N=34	N=22	N=147	N=20	N=16	N=23
Emotionally reactive	1 (ref)	1.15 (0.76, 1.76)	1.15 (0.80, 1.65)	1.28 (0.86, 1.91)	1 (ref)	1.07 (0.72, 1.60)	0.92 (0.58, 1.46)	0.96 (0.64, 1.46)
Anxious/depressed	1 (ref)	1.05 (0.75, 1.49)	1.17 (0.88, 1.56)	1.10 (0.79, 1.54)	1 (ref)	1.24 (0.90, 1.71)	0.92 (0.62, 1.35)	1.14 (0.82, 1.59)
Somatic complaints	1 (ref)	0.96 (0.69, 1.34)	1.00 (0.75, 1.32)	1.25 (0.93, 1.68)	1 (ref)	1.20 (0.87, 1.64)	0.98 (0.69, 1.41)	1.05 (0.76, 1.44)
Withdrawn	1 (ref)	1.21 (0.76, 1.92)	1.05 (0.69, 1.60)	0.72 (0.43, 1.21)	1 (ref)	0.97 (0.59, 1.60)	0.91 (0.53, 1.57)	0.88 (0.52, 1.50)
Sleep problems	1 (ref)	1.36 (0.94, 1.96)	1.13 (0.81, 1.57)	1.18 (0.81, 1.71)	1 (ref)	1.10 (0.77, 1.57)	1.06 (0.71, 1.60)	1.06 (0.73, 1.55)
Attention problems	1 (ref)	**0.60 (0.38, 0.93)**[*]	0.92 (0.66, 1.27)	0.88 (0.60, 1.27)	1 (ref)	1.21 (0.84, 1.75)	0.78 (0.48, 1.28)	0.92 (0.61, 1.38)
Aggressive behaviors	1 (ref)	1.01 (0.75, 1.36)	1.03 (0.79, 1.33)	1.27 (0.95, 1.70)	1 (ref)	1.07 (0.78, 1.47)	1.10 (0.77, 1.55)	0.93 (0.68, 1.29)
Internalizing behaviors	1 (ref)	1.08 (0.81, 1.44)	1.09 (0.85, 1.40)	1.14 (0.86, 1.51)	1 (ref)	1.16 (0.89, 1.53)	0.93 (0.69, 1.26)	1.01 (0.76, 1.33)
Externalizing behaviors	1 (ref)	0.94 (0.71, 1.26)	1.01 (0.79, 1.29)	1.21 (0.92, 1.60)	1 (ref)	1.09 (0.81, 1.47)	1.05 (0.75, 1.47)	0.94 (0.69, 1.27)

Abbreviations: CBCL, Child Behavior Checklist

Adjusted for paternal age, paternal body mass index, paternal education, paternal smoking, maternal age, parity, maternal depressive symptoms during pregnancy, maternal preconception folic acid supplements, multivitamin supplements during pregnancy, and gestational weeks

[*] Statistically significant differences (*P*-value < 0.05)

Table S5. Associations between preconceptional paternal alcohol consumption and CBCL raw scores at 2, 4 and 6 years of age in children without maternal passive smoking

CBCL Raw Scores	All subjects	Boys	Girls
	**Adjusted RR (95% CI)[b]**	**Adjusted RR (95% CI)[c]**	**Adjusted RR (95% CI)[c]**
**Children 2 years of age**	N=283	N=160	N=123
Emotionally reactive	1.14 (0.88, 1.48)	1.14 (0.81, 1.62)	1.19 (0.78, 1.83)
Anxious/depressed	1.04 (0.84, 1.28)	1.05 (0.78, 1.41)	1.11 (0.79, 1.55)
Somatic Complaints	0.95 (0.77, 1.16)	0.92 (0.70, 1.20)	1.05 (0.75, 1.48)
Withdrawn	0.91 (0.67, 1.23)	0.80 (0.53, 1.21)	1.28 (0.81, 2.01)
Sleep problems	1.18 (0.94, 1.47)	1.07 (0.78, 1.45)	**1.52 (1.06, 2.17)***
Attention problems	0.89 (0.70, 1.14)	0.76 (0.54, 1.07)	1.32 (0.88, 1.97)
Aggressive behaviors	1.02 (0.84, 1.24)	1.00 (0.78, 1.27)	1.19 (0.85, 1.66)
Internalizing	1.02 (0.86, 1.22)	0.99 (0.78, 1.26)	1.15 (0.86, 1.54)
Externalizing	1.01 (0.84, 1.21)	0.96 (0.76, 1.21)	1.21 (0.90, 1.65)
**Children 4 years of age**	N=386	N=218	N=168
Emotionally reactive	**1.28 (1.04, 1.58)***	1.23 (0.92, 1.65)	**1.35 (0.98, 1.86)#**
Anxious/depressed	**1.26 (1.04, 1.52)***	1.01 (0.77, 1.32)	**1.66 (1.26, 2.20)***
Somatic Complaints	1.02 (0.85, 1.24)	1.06 (0.83, 1.36)	0.98 (0.72, 1.33)
Withdrawn	0.97 (0.73, 1.29)	0.85 (0.59, 1.23)	1.31 (0.83, 2.07)
Sleep problems	1.16 (0.94, 1.44)	1.10 (0.82, 1.48)	**1.39 (1.01, 1.92)***
Attention problems	1.07 (0.88, 1.29)	1.05 (0.81, 1.36)	1.11 (0.81, 1.52)
Aggressive behaviors	1.08 (0.91, 1.29)	1.17 (0.94, 1.46)	1.02 (0.76, 1.37)
Internalizing	**1.16 (0.99, 1.36)#**	1.06 (0.86, 1.31)	**1.34 (1.04, 1.73)***
Externalizing	1.08 (0.91, 1.27)	1.13 (0.92, 1.40)	1.04 (0.80, 1.36)
**Children 6 years of age**	N=325	N=188	N=137
Anxious/depressed	**1.49 (1.12, 1.97)***	1.25 (0.85, 1.83)	**1.81 (1.17, 2.82)***
Withdrawn/depressed	0.95 (0.71, 1.27)	0.88 (0.60, 1.31)	0.97 (0.60, 1.57)
Somatic complaints	**1.46 (1.00, 2.14)#**	1.53 (0.89, 2.62)	1.23 (0.68, 2.22)
Social problems	1.18 (0.93, 1.50)	1.06 (0.76, 1.46)	1.32 (0.93, 1.88)
Thought problems	**1.35 (1.05, 1.72)***	1.15 (0.81, 1.62)	**1.62 (1.11, 2.37)***
Attention problems	**1.24 (1.03, 1.50)***	**1.27 (0.99, 1.64)#**	1.21 (0.90, 1.62)
Rule-breaking	**1.33 (1.06, 1.67)***	**1.57 (1.18, 2.09)***	0.79 (0.51, 1.23)
Aggressive behaviors	1.20 (0.95, 1.52)	**1.32 (0.98, 1.76)#**	0.92 (0.61, 1.39)
Internalizing	**1.29 (1.01, 1.64)***	1.14 (0.83, 1.55)	**1.43 (0.96, 2.13)#**
Externalizing	**1.24 (1.00, 1.54)#**	**1.38 (1.05, 1.82)***	0.92 (0.64, 1.33)

Abbreviations：CBCL，Child Behavior Checklist

[b] Adjusted for paternal age, paternal body mass index, paternal education, paternal smoking, maternal age, parity, maternal depressive symptoms during pregnancy, maternal preconception folic acid supplements, multivitamin supplements during pregnancy, gestational weeks, and sex.

[c] Adjusted for all potential confounding variables above except sex.

* Statistically significant differences (p-value < 0.05), # p-value < 0.10

Table S6. Associations between preconceptional paternal alcohol consumption (yes/no) and CBCL raw scores for children at 2, 4 and 6 years of age among mothers with education at college or university.

CBCL Raw Scores	All	Boy	Girl
	**Adjusted RR (95% CI)[b]**	**Adjusted RR (95% CI)[c]**	**Adjusted RR (95% CI)[c]**
**Children at 2 years of age**	N=351	N=191	N=159
Emotionally reactive	1.17 (0.95, 1.43)	1.25 (0.94, 1.65)	0.98 (0.72, 1.34)
Anxious/depressed	1.04 (0.89, 1.23)	0.99 (0.79, 1.24)	1.15 (0.90, 1.46)
Somatic Complaints	1.08 (0.91, 1.27)	1.03 (0.81, 1.30)	1.24 (0.98, 1.58)
Withdrawn	1.01 (0.78, 1.30)	1.07 (0.74, 1.55)	0.90 (0.63, 1.30)
Sleep problems	1.13 (0.92, 1.37)	1.27 (0.96, 1.69)	1.04 (0.77, 1.39)
Attention problems	0.84 (0.69, 1.02)	0.87 (0.66, 1.15)	0.81 (0.59, 1.10)
Aggressive behaviors	1.09 (0.93, 1.28)	1.10 (0.88, 1.38)	1.04 (0.81, 1.34)
Internalizing behaviors	1.07 (0.93, 1.24)	1.08 (0.88, 1.32)	1.09 (0.89, 1.35)
Externalizing behaviors	1.05 (0.90, 1.22)	1.06 (0.85, 1.31)	1.01 (0.80, 1.28)
**Children at 4 years of age**	N=449	N=249	N=200
Emotionally reactive	1.18 (0.97, 1.43)	1.13 (0.87, 1.47)	1.24 (0.93, 1.66)
Anxious/depressed	**1.20 (1.01, 1.42)***	1.09 (0.86, 1.38)	**1.34 (1.06, 1.71)***
Somatic Complaints	**1.21 (1.04, 1.41)***	**1.20 (0.98, 1.47)#**	**1.25 (1.00, 1.57)***
Withdrawn	1.04 (0.83, 1.30)	0.98 (0.72, 1.34)	1.15 (0.83, 1.58)
Sleep problems	**1.25 (1.06, 1.49)***	**1.23 (0.98, 1.54)#**	**1.32 (1.03, 1.70)***
Attention problems	1.04 (0.88, 1.22)	1.11 (0.89, 1.39)	0.95 (0.73, 1.24)
Aggressive behaviors	1.12 (0.95, 1.31)	1.17 (0.94, 1.44)	1.07 (0.84, 1.36)
Internalizing behaviors	**1.18 (1.02, 1.35)***	1.13 (0.94, 1.37)	**1.25 (1.01, 1.55)***
Externalizing behaviors	1.10 (0.95, 1.27)	1.15 (0.95, 1.40)	1.04 (0.84, 1.30)
**Children at 6 years of age**	N=386	N=219	N=167
Anxious/depressed	1.13 (0.89, 1.43)	0.96 (0.68, 1.35)	1.24 (0.89, 1.74)
Withdrawn/depressed	0.92 (0.71, 1.18)	0.80 (0.56, 1.15)	0.98 (0.69, 1.39)
Somatic complaints	**1.41 (1.02, 1.95)***	**1.69 (1.10, 2.59)***	1.16 (0.70, 1.90)
Social problems	1.14 (0.94, 1.39)	1.06 (0.82, 1.39)	1.14 (0.86, 1.50)
Thought problems	**1.20 (0.98, 1.48)#**	1.14 (0.86, 1.51)	1.21 (0.89, 1.65)
Attention problems	1.12 (0.95, 1.32)	1.21 (0.95, 1.54)	1.00 (0.79, 1.26)
Rule-breaking behaviors	**1.19 (0.98, 1.45)#**	**1.28 (1.01, 1.62)***	1.05 (0.74, 1.49)
Aggressive behaviors	1.10 (0.91, 1.33)	1.18 (0.92, 1.50)	0.96 (0.70, 1.32)
Internalizing behaviors	1.12 (0.92, 1.36)	1.01 (0.77, 1.32)	1.17 (0.86, 1.59)
Externalizing behaviors	1.13 (0.94, 1.35)	1.21 (0.96, 1.51)	1.00 (0.74, 1.34)

Abbreviations: CBCL, Child Behavior Checklist

[b]Adjusted for paternal age, paternal body mass index, paternal education, paternal smoking, maternal age, parity, maternal depressive symptoms during pregnancy, maternal preconception folic acid supplements, multivitamin supplements during pregnancy, gestational weeks, and sex.

[c] Adjusted for all potential confounding variables above except sex.

* Statistically significant differences (P-value < 0.05), #P-value < 0.10.

Table S7. Associations between preconceptional paternal alcohol consumption (yes/no) and CBCL raw scores for children at 2, 4 and 6 years of age among fathers with education at college or university.

CBCL Raw Scores	All	Boy	Girl
	**Adjusted RR (95% CI)[b]**	**Adjusted RR (95% CI)[c]**	**Adjusted RR (95% CI)[c]**
**Children at 2 years of age**	N=329	N=189	N=139
Emotionally reactive	**1.21 (0.97, 1.51)[#]**	1.18 (0.84, 1.66)	1.14 (0.83, 1.57)
Anxious/depressed	1.15 (0.96, 1.37)	1.11 (0.85, 1.45)	1.20 (0.94, 1.53)
Somatic Complaints	1.06 (0.89, 1.27)	1.10 (0.85, 1.41)	1.13 (0.87, 1.48)
Withdrawn	1.11 (0.86, 1.45)	1.14 (0.77, 1.68)	1.10 (0.74, 1.62)
Sleep problems	**1.22 (0.99, 1.49)[#]**	**1.39 (1.03, 1.87)[*]**	1.18 (0.88, 1.59)
Attention problems	0.90 (0.73, 1.11)	0.90 (0.67, 1.21)	0.91 (0.66, 1.27)
Aggressive behaviors	1.12 (0.95, 1.32)	1.11 (0.87, 1.42)	1.11 (0.86, 1.43)
Internalizing behaviors	1.14 (0.98, 1.32)	1.13 (0.90, 1.42)	1.17 (0.94, 1.44)
Externalizing behaviors	1.09 (0.93, 1.28)	1.08 (0.85, 1.36)	1.09 (0.86, 1.40)
**Children at 4 years of age**	N=433	N=250	N=183
Emotionally reactive	**1.19 (0.98, 1.45)[#]**	1.17 (0.89, 1.54)	1.20 (0.90, 1.59)
Anxious/depressed	**1.26 (1.07, 1.50)[*]**	1.21 (0.94, 1.56)	**1.35 (1.08, 1.70)[*]**
Somatic Complaints	**1.12 (0.97, 1.31)[#]**	**1.19 (0.97, 1.47)[#]**	1.04 (0.83, 1.29)
Withdrawn	1.07 (0.85, 1.34)	1.08 (0.77, 1.52)	1.15 (0.85, 1.55)
Sleep problems	**1.18 (0.98, 1.41)[#]**	1.23 (0.95, 1.58)	1.21 (0.94, 1.55)
Attention problems	1.07 (0.89, 1.27)	1.09 (0.86, 1.38)	1.01 (0.78, 1.32)
Aggressive behaviors	1.13 (0.96, 1.32)	1.18 (0.95, 1.47)	1.07 (0.85, 1.36)
Internalizing behaviors	**1.17 (1.02, 1.35)[*]**	**1.19 (0.97, 1.46)[#]**	1.17 (0.96, 1.43)
Externalizing behaviors	1.12 (0.96, 1.29)	1.17 (0.95, 1.43)	1.06 (0.85, 1.31)
**Children at 6 years of age**	N=378	N=224	N=154
Anxious/depressed	1.16 (0.91, 1.48)	1.04 (0.73, 1.50)	1.31 (0.93, 1.84)
Withdrawn/depressed	0.80 (0.62, 1.04)	**0.67 (0.45, 0.97)[*]**	0.97 (0.68, 1.39)
Somatic complaints	**1.36 (0.98, 1.89)[#]**	**1.86 (1.19, 2.90)[*]**	0.95 (0.59, 1.53)
Social problems	1.10 (0.90, 1.35)	1.11 (0.84, 1.47)	1.07 (0.80, 1.44)
Thought problems	1.14 (0.92, 1.41)	1.14 (0.85, 1.53)	1.12 (0.81, 1.55)
Attention problems	1.07 (0.90, 1.27)	1.14 (0.90, 1.44)	1.02 (0.80, 1.29)
Rule-breaking behaviors	**1.23 (1.00, 1.50)[*]**	**1.27 (0.99, 1.64)[#]**	1.11 (0.79, 1.57)
Aggressive behaviors	1.01 (0.82, 1.23)	1.11 (0.85, 1.43)	0.88 (0.64, 1.23)
Internalizing behaviors	1.07 (0.88, 1.32)	1.01 (0.77, 1.34)	1.14 (0.85, 1.53)
Externalizing behaviors	1.07 (0.89, 1.29)	1.15 (0.91, 1.47)	0.95 (0.71, 1.28)

Abbreviations: CBCL, Child Behavior Checklist

[b]Adjusted for paternal age, paternal body mass index, paternal smoking, maternal age, parity, maternal depressive symptoms during pregnancy, maternal preconception folic acid supplements, multivitamin supplements during pregnancy, gestational weeks, and sex.

[c]Adjusted for all potential confounding variables above

* Statistically significant differences (P-value < 0.05), [#]P-value < 0.10.

Table S8. Associations between preconceptional paternal alcohol consumption (yes/no) and CBCL raw scores for children at 2, 4 and 6 years of age among fathers with normal BMI (BMI18.5~24 kg/m^2).

**CBCL Raw Scores**	**All**	**Boy**	**Girl**
	**Adjusted RR (95% CI)[b]**	**Adjusted RR (95% CI)[c]**	**Adjusted RR (95% CI)[c]**
**Children at 2 years of age**	N=280	N=145	N=134
Emotionally reactive	1.14 (0.91, 1.42)	1.24 (0.89, 1.71)	1.02 (0.74, 1.41)
Anxious/depressed	1.06 (0.87, 1.27)	1.11 (0.83, 1.47)	1.09 (0.84, 1.43)
Somatic Complaints	1.07 (0.90, 1.27)	1.09 (0.84, 1.42)	1.04 (0.81, 1.33)
Withdrawn	1.02 (0.78, 1.33)	1.20 (0.80, 1.81)	1.01 (0.70, 1.47)
Sleep problems	1.06 (0.86, 1.31)	1.18 (0.85, 1.64)	1.11 (0.84, 1.47)
Attention problems	0.85 (0.68, 1.06)	0.69 (0.50, 0.97)	1.04 (0.75, 1.45)
Aggressive behaviors	1.09 (0.92, 1.29)	1.16 (0.89, 1.50)	1.07 (0.84, 1.37)
Internalizing behaviors	1.08 (0.93, 1.25)	1.16 (0.92, 1.45)	1.06 (0.85, 1.3)
Externalizing behaviors	1.06 (0.90, 1.24)	1.07 (0.83, 1.38)	1.08 (0.86, 1.36)
**Children at 4 years of age**	N=371	N=204	N=167
Emotionally reactive	1.21 (0.98, 1.49)	**1.37 (1.02, 1.83)***	1.08 (0.79, 1.47)
Anxious/depressed	**1.22 (1.03, 1.46)***	**1.26 (0.98, 1.62)#**	**1.25 (0.97, 1.61)**
Somatic Complaints	0.99 (0.84, 1.16)	1.11 (0.89, 1.39)	0.84 (0.66, 1.08)
Withdrawn	1.02 (0.79, 1.31)	1.18 (0.82, 1.70)	0.99 (0.70, 1.40)
Sleep problems	**1.18 (0.97, 1.43)#**	1.15 (0.89, 1.49)	1.23 (0.90, 1.67)
Attention problems	1.01 (0.83, 1.21)	1.08 (0.84, 1.39)	0.87 (0.65, 1.18)
Aggressive behaviors	1.09 (0.91, 1.29)	**1.23 (0.97, 1.57)#**	1.01 (0.78, 1.31)
Internalizing behaviors	1.11 (0.96, 1.30)	**1.23 (0.99, 1.54)#**	1.04 (0.83, 1.29)
Externalizing behaviors	1.07 (0.91, 1.25)	1.19 (0.96, 1.49)	0.98 (0.77, 1.25)
**Children at 6 years of age**	N=316	N=179	N=137
Anxious/depressed	**1.46 (1.11, 1.91)***	1.33 (0.93, 1.90)	1.41 (0.92, 2.17)
Withdrawn/depressed	0.88 (0.68, 1.12)	0.80 (0.57, 1.13)	0.86 (0.59, 1.25)
Somatic complaints	1.37 (0.96, 1.97)	1.46 (0.90, 2.38)	1.19 (0.70, 2.04)
Social problems	**1.21 (0.99, 1.47)#**	1.18 (0.90, 1.55)	1.12 (0.85, 1.49)
Thought problems	1.15 (0.91, 1.44)	1.01 (0.74, 1.38)	1.23 (0.87, 1.74)
Attention problems	1.07 (0.89, 1.28)	1.17 (0.91, 1.50)	0.91 (0.70, 1.19)
Rule-breaking behaviors	1.12 (0.90, 1.40)	1.12 (0.86, 1.47)	1.06 (0.72, 1.57)
Aggressive behaviors	1.03 (0.84, 1.27)	1.10 (0.84, 1.44)	0.88 (0.62, 1.26)
Internalizing behaviors	**1.22 (0.99, 1.51)#**	1.11 (0.85, 1.46)	1.16 (0.82, 1.64)
Externalizing behaviors	1.06 (0.87, 1.29)	1.11 (0.86, 1.43)	0.94 (0.67, 1.31)

Abbreviations: CBCL, Child Behavior Checklist

[b]Adjusted for paternal age, paternal education, paternal smoking, maternal age, parity, maternal depressive symptoms during pregnancy, maternal preconception folic acid supplements, multivitamin supplements during pregnancy, gestational weeks, and sex.

[c]Adjusted for all potential confounding variables above

* Statistically significant differences (P-value < 0.05), #P-value < 0.10.

Table S9. Associations between preconceptional paternal alcohol consumption (yes/no) and CBCL raw scores for children at 2, 4 and 6 years of age among mothers with normal pre-pregnancy BMI (BMI 18.5~24 kg/m^2).

CBCL Raw Scores	All	Boy	Girl
	**Adjusted RR (95% CI)[b]**	**Adjusted RR (95% CI)[c]**	**Adjusted RR (95% CI)[c]**
**Children at 2 years of age**	N=356	N=182	N=172
Emotionally reactive	1.12 (0.91, 1.38)	1.26 (0.91, 1.74)	0.97 (0.72, 1.31)
Anxious/depressed	1.03 (0.87, 1.22)	1.02 (0.79, 1.32)	1.09 (0.85, 1.39)
Somatic Complaints	1.00 (0.85, 1.18)	0.96 (0.75, 1.24)	1.14 (0.90, 1.45)
Withdrawn	0.93 (0.71, 1.21)	0.93 (0.61, 1.40)	0.97 (0.66, 1.41)
Sleep problems	1.05 (0.87, 1.28)	1.13 (0.86, 1.49)	1.00 (0.75, 1.33)
Attention problems	0.87 (0.71, 1.07)	0.80 (0.58, 1.09)	0.99 (0.73, 1.34)
Aggressive behaviors	1.04 (0.89, 1.22)	1.06 (0.83, 1.35)	1.01 (0.80, 1.26)
Internalizing behaviors	1.03 (0.90, 1.18)	1.05 (0.84, 1.31)	1.06 (0.87, 1.29)
Externalizing behaviors	1.02 (0.87, 1.18)	1.02 (0.80, 1.29)	1.01 (0.81, 1.24)
**Children at 4 years of age**	N=471	N=255	N=216
Emotionally reactive	1.01 (0.83, 1.22)	0.96 (0.74, 1.25)	0.98 (0.74, 1.30)
Anxious/depressed	1.05 (0.89, 1.24)	1.01 (0.80, 1.27)	1.06 (0.83, 1.35)
Somatic Complaints	1.02 (0.88, 1.19)	1.19 (0.97, 1.46)	0.86 (0.68, 1.10)
Withdrawn	0.82 (0.64, 1.06)	0.86 (0.61, 1.21)	0.85 (0.59, 1.23)
Sleep problems	1.13 (0.95, 1.35)	1.16 (0.91, 1.48)	1.07 (0.83, 1.39)
Attention problems	0.91 (0.76, 1.09)	0.87 (0.68, 1.12)	0.95 (0.73, 1.25)
Aggressive behaviors	0.94 (0.80, 1.10)	1.00 (0.81, 1.25)	0.85 (0.67, 1.07)
Internalizing behaviors	1.00 (0.86, 1.15)	1.03 (0.85, 1.25)	0.95 (0.77, 1.18)
Externalizing behaviors	0.93 (0.81, 1.08)	0.97 (0.79, 1.19)	0.87 (0.70, 1.08)
**Children at 6 years of age**	N=395	N=223	N=172
Anxious/depressed	1.10 (0.85, 1.43)	0.99 (0.67, 1.46)	1.15 (0.81, 1.63)
Withdrawn/depressed	0.79 (0.62, 1.01)	0.75 (0.53, 1.05)	0.87 (0.61, 1.25)
Somatic complaints	**1.41 (1.02, 1.94)***	1.60 (1.04, 2.46)	1.21 (0.75, 1.95)
Social problems	1.04 (0.85, 1.27)	1.00 (0.76, 1.31)	1.06 (0.78, 1.43)
Thought problems	1.16 (0.93, 1.45)	1.14 (0.85, 1.55)	1.12 (0.80, 1.55)
Attention problems	0.98 (0.82, 1.16)	1.02 (0.80, 1.30)	0.92 (0.72, 1.18)
Rule-breaking behaviors	1.11 (0.90, 1.37)	1.11 (0.85, 1.46)	1.05 (0.77, 1.45)
Aggressive behaviors	1.01 (0.83, 1.23)	1.07 (0.82, 1.38)	0.85 (0.62, 1.16)
Internalizing behaviors	1.03 (0.84, 1.27)	0.98 (0.74, 1.31)	1.06 (0.78, 1.44)
Externalizing behaviors	1.06 (0.88, 1.27)	1.09 (0.85, 1.39)	0.94 (0.70, 1.24)

Abbreviations: CBCL, Child Behavior Checklist

[b]Adjusted for paternal age, paternal body mass index, paternal education, paternal smoking, maternal age, parity, maternal depressive symptoms during pregnancy, maternal preconception folic acid supplements, multivitamin supplements during pregnancy, gestational weeks, and sex.

[c] Adjusted for all potential confounding variables above except sex.

* Statistically significant differences (P-value < 0.05), [#]P-value < 0.10.
